# A case of aortitis during cisplatin-based chemotherapy for cervical cancer

**DOI:** 10.1259/bjrcr.20180054

**Published:** 2018-09-08

**Authors:** Katharine Webb, Vineet Prakash, Othman Kirresh, Alexandra Stewart

**Affiliations:** 1 St Luke's Cancer Centre, Royal Surrey County Hospital, Guildford, UK; 2 Faculty of Health and Medical Sciences, University of Surrey, Guildford, UK

## Abstract

A case of aortitis in a patient undergoing adjuvant cisplatin and topotecan chemotherapy for cervical cancer following presentation with pyrexia of unknown origin and raised inflammatory markers is presented. Although many chemotherapy agents are known to cause small vessel vasculitis and there are several reported cases of large vessel vasculitis following gemcitabine chemotherapy, there is only one previously described case of aortitis following cisplatin administration. This case is presented with corresponding CT and ^18^F-FDG PET-CT imaging with discussion of the literature regarding vasculitis and chemotherapy.

## Clinical presentation

A 51-year-old female was undergoing adjuvant cisplatin and topotecan chemotherapy (three-weekly cycles of cisplatin 50 mg m^−^^2^ i.v. on Day 1, and topotecan 0.75 mg m^−^^2^ day^−1^ i.v. on Day 1, 2 and 3) following bilateral salpingo-oopherectomy and subsequent chemoradiotherapy (with six doses of concurrent cisplatin 40 mg m^−^^2^ i.v. given once weekly) for a FIGO Stage IVB cervical cancer (staging due to deposits on the peritoneal surface of the ovaries). 10 days following the third cycle, the patient was admitted with febrile neutropaenia with no localising symptoms or signs of infection. She was commenced on broad spectrum antibiotics. The neutropaenia resolved after 3 days, however, the C-reactive protein (CRP) remained very elevated, ranging between 200 and 300 mg l^−1^ and the patient consistently spiked temperatures >38 °C for the next 10 days. A chest X-ray was unremarkable and multiple (>12) blood cultures were negative, as well as multiple stool and urine cultures. A CT thorax, abdomen and pelvis on Day 5 of admission revealed no source of infection.

**Figure 1.**
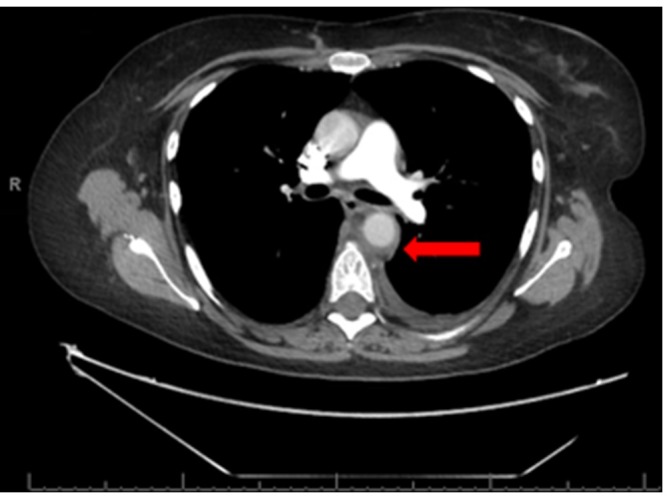
Axial CT with i.v. contrast. Abnormal circumferential mural thickening is seen in the descending thoracic aorta (red arrow). Also, a small left pleural effusion is noted.

**Figure 2.**
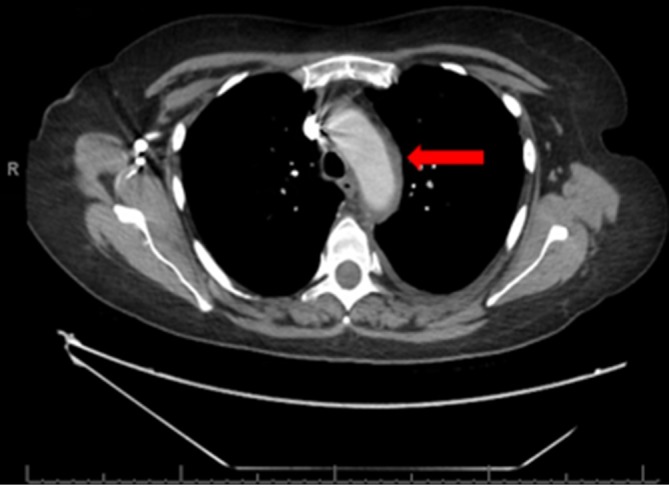
Axial CT with i.v. contrast. Abnormal circumferential mural thickening is seen in the aortic arch (red arrow).

Due to the patient complaining of mild left posterior back pain, associated with a slight non-productive cough, a CT pulmonary angiogram was performed on Day 9 of admission to rule out a pulmonary embolus. Although this showed no PE, it revealed periaortic stranding and low density soft tissue extending from the aortic arch along the descending thoracic aorta, which, in combination with the high temperatures and raised inflammatory markers, was suggestive of aortitis (Figures 1 and 2). Aortic calibre was normal, and there was nothing on imaging to suggest the aortitis was due to an infective cause. On retrospective review, this thickening was subtly present on the previous CT imaging from 4 days earlier.

**Figure 3.**
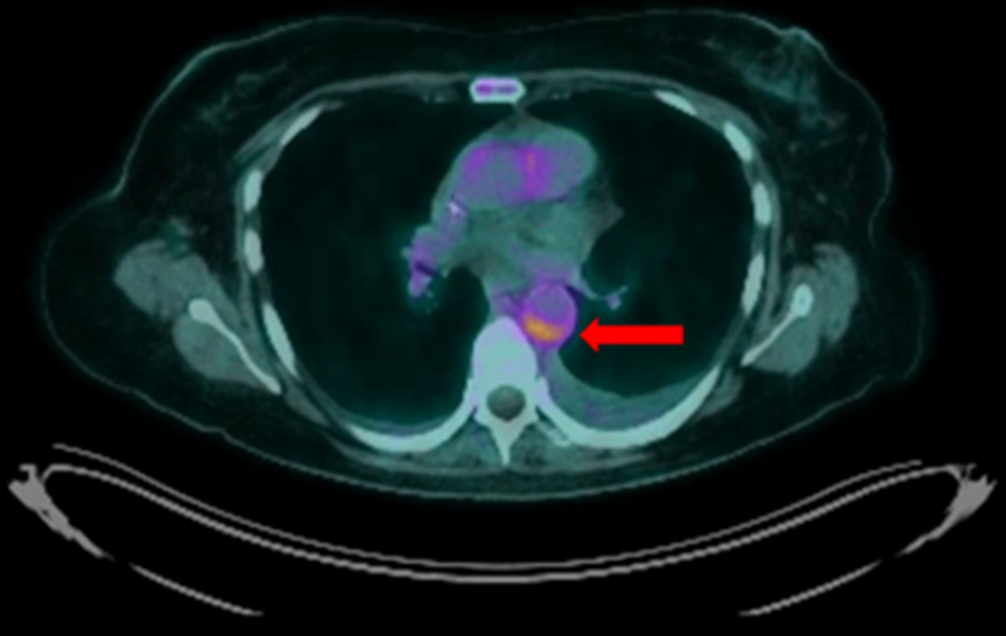
Fused axial ^18^F-FDG PET-CT shows increased FDG avidity in the aortic wall corresponding to the mural thickening (red arrow). FDG, fludeoxyglucose.

**Figure 4.**
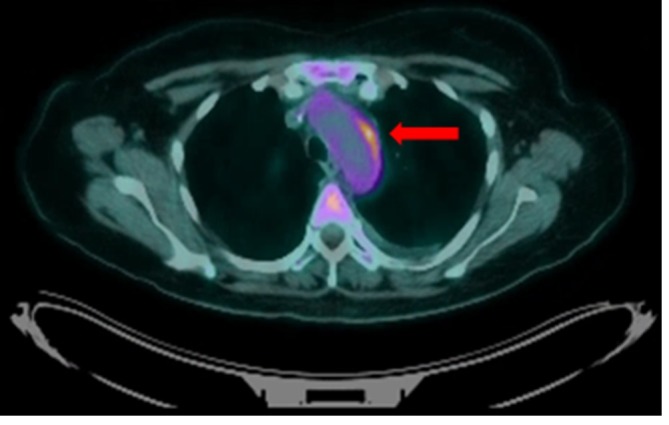
Fused axial ^18^F-FDG PET-CT shows increased FDG avidity in the aortic wall corresponding to the mural thickening (red arrow). FDG, fludeoxyglucose.

**Figure 5.**
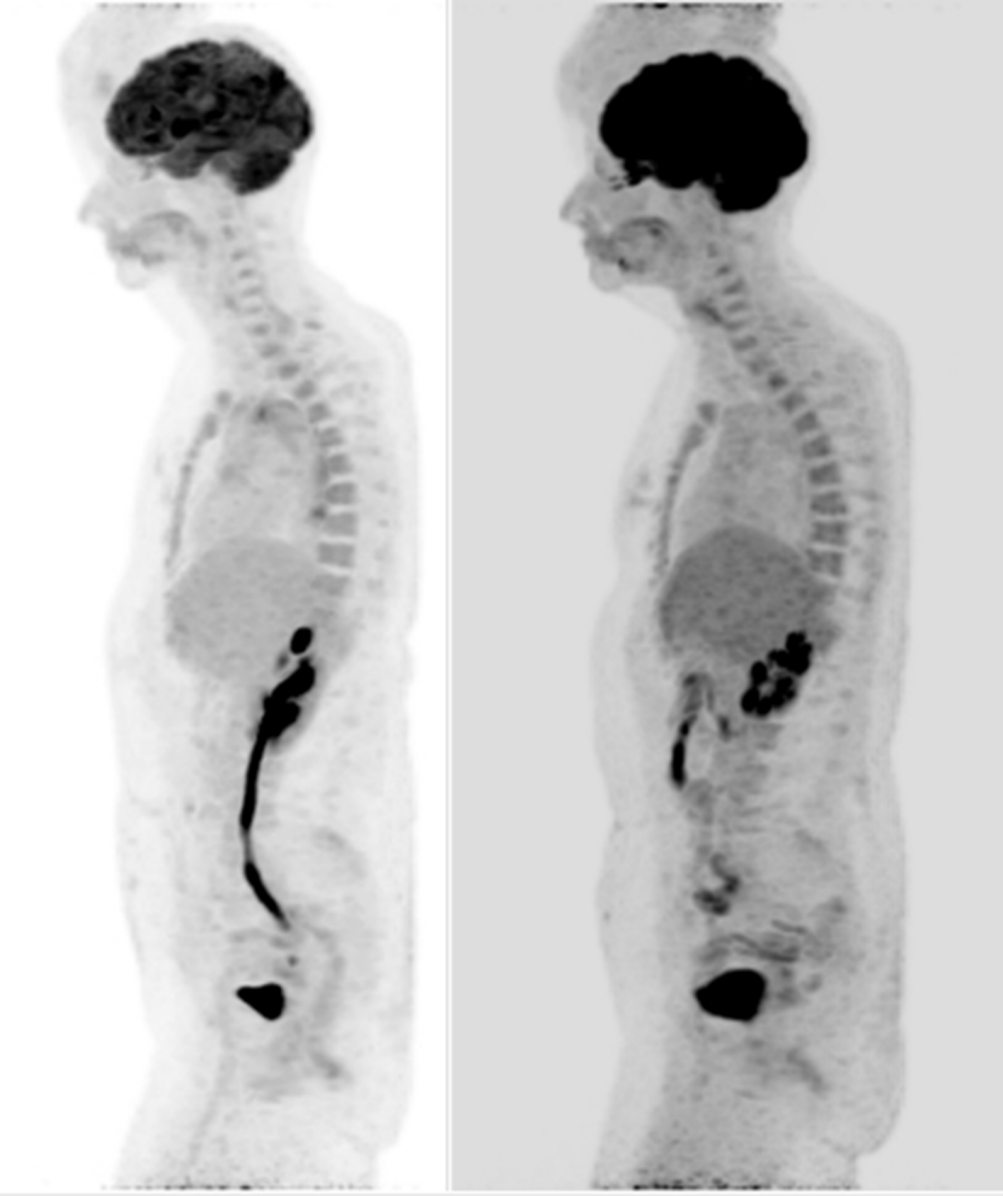
(Left image) MIP sagittal ^18^F-FDG PET-CT shows increased FDG avidity in the aortic wall corresponding to the mural thickening. (Right image) Post therapy MIP Sagittal ^18^F-FDG PET-CT shows complete resolution of the abnormal metabolic activity in keeping with a metabolic response. MIP, maximum intensity projection.

An ^18^F-fludeoxyglucose (FDG) PET-CT scan was performed to help confirm the diagnosis, as well as rule out occult infection or cervical cancer recurrence. This revealed focal regions of increased tracer uptake around the aortic arch, in relation to the posterior wall of the descending thoracic aorta, consistent with regions of active large vessel vasculitis. There was no uptake apparent elsewhere, including in the treated area around the cervix, in keeping with a complete metabolic response (Figures 3–5). An ^18^F-FDG PET-CT scan performed at diagnosis (several months prior to this presentation) showed no tracer uptake in the aortic region.

The patient was commenced on 40 mg once daily of Prednisolone and within 24 h of starting steroids, the fevers resolved and the CRP started to fall. Within 2 weeks, the CRP had fallen to <4. Adjuvant chemotherapy was stopped. Repeat ^18^F-FDG PET-CT scanning 3 months later demonstrated complete resolution of the changes.

## Imaging

Figures 1–5 for CT and ^18^F-FDG PET-CT images.

### Possible aetiology

Full history revealed no symptoms of underlying vasculitic conditions or systemic inflammatory disorders, with no headache, scalp tenderness, jaw or arm claudication, visual symptoms, joint pains, rashes or ENT symptoms.

Clinical examination revealed no murmurs, bruits, temporal artery tenderness, scalp tenderness or joint synovitis, no rashes or connective tissue features, unremarkable blood pressure which was equal in both arms, no radiofemoral or radioradial delay, and no nail fold infarcts. As a result, there were no obvious clinical features to suggest either of the two most common non-infective causes of aortitis; Takayasu’s arteritis or Giant Cell Arteritis.^1^ Additionally, an ANCA was negative and immunoglobulins unremarkable. Erythrocyte sedimentation rate was raised as expected at 140.

Another common cause of aortitis is infective,^2^ however, the patient had no symptoms or signs of localised infection, did not respond to broad-spectrum antibiotics and had multiple negative blood, urine and stool cultures. Neither CT pulmonary angiogram nor PET revealed any features suggesting an underlying infective cause of the aortitis. Additionally, the fevers settled rapidly with the introduction of steroids alone, which indicates an inflammatory cause.

As a result, the most likely culprits are the chemotherapy agents—cisplatin or topotecan.

## Discussion

Although an association between vasculitis and several chemotherapy agents has been reported, no reports of large vessel vasculitis following intravenous administration of cisplatin or topotecan have been reported. Cisplatin has been described in association with large vessel vasculitis following intra-arterial administration. Tanaka et al^3^ described a case of aortitis following intra-arterial infusion of cisplatin-based chemotherapy for cervical cancer. The tip of the catheter was placed in the abdominal aorta and aortitis occurred. A chemical aortitis, possibly as a result of catheter tip displacement and flow to the vasa vasorum, was felt to be the likely cause of this.

There have been four case reports describing large vessel vasculitis (aortitis or carotiditis) in patients undergoing gemcitabine-based chemotherapies.^4–7^ In at least one case,^6^ the co-administered drug was carboplatin and one patient had recently received cisplatin concurrently with radiotherapy for bladder cancer. In all cases, the condition resolved with high dose prolonged steroids and cessation of chemotherapy agents.

Gemcitabine, methotrexate and vincristine have been implicated in causing small vessel, or leukocytoclastic, vasculitis.^8^ Three cases of leukocytoclastic vasculitis in patients receiving oxaliplatin (with 5-fluorouracil) have been described in the literature.^9,10^ There have also been multiple reports of small and medium vessel vasculitis following gemcitabine in combination with cisplatin ± taxanes.^6,11,12^ Although gemcitabine as monotherapy (or in combination with a taxane) has been implicated in several of these cases, in the majority of cases, the chemotherapy regimen included cisplatin or carboplatin (8 out of 11 reported cases).

A case report by Schmorl et al^13^ implicated the combination of gemcitabine and cisplatin in cerebral vasculitis in a 50-year-old lady with bladder cancer. Although the temporal relationship suggested gemcitabine was the causative agent, the patient had also recently received cisplatin. Of note, in the cisplatin summary of product characteristics, cerebral arteritis is listed in the table of adverse drug events reported during clinical or post-marketing experience, with the frequency listed as “not known”.

Cisplatin is well-known to have significant vascular toxicity^14–19^ causing thromboembolic disease, including thrombosis of the major vessels, including the aorta,^20–27^ thrombotic microangiopathy, myocardial infarction, cerebrovascular accidents, hypertension, and Raynaud’s phenomenon. There are multiple putative mechanisms underlying these effects, including changes in platelet aggregation and activation, endothelial disruption, vasospasm, hypomagnesaemia and increased vasoreactivity. Although vasospasm is likely the primary mechanism of action in Raynaud’s phenomenon, a study by Vogelzang et al^28^ revealed diffuse arteritis on arteriography in two patients with Raynaud’s following chemotherapy for testicular cancer in a regimen that included cisplatin.

There are no cases in the literature to suggest that topotecan or other topoisomerase inhibitors are implicated in causing small or large vessel vasculitis.

It is likely, therefore, that this patient’s aortitis was caused by recent cisplatin chemotherapy.

## Learning points

1. Consider aortitis in patients on cisplatin or other chemotherapy agents presenting constitutionally unwell with pyrexia and raised inflammatory markers of undetermined cause, in order to expedite diagnosis of this potentially life-threatening but eminently treatable condition.
